# A meta-analysis of the weight of advice in decision-making

**DOI:** 10.1007/s12144-022-03573-2

**Published:** 2022-08-09

**Authors:** Phoebe E. Bailey, Tarren Leon, Natalie C. Ebner, Ahmed A. Moustafa, Gabrielle Weidemann

**Affiliations:** 1 Graduate School of Health, University of Technology Sydney, P. O. Box 123, Broadway, NSW 2007, Australia; 2 Department of Psychology, University of Florida, Gainesville, FL, USA; 3 School of Psychology, Bond University, Robina, Australia; 4 School of Psychology, Western Sydney University, Penrith, Australia

**Keywords:** Advice-taking, Estimation, Meta-analysis, Egocentric discounting, Decision-making

## Abstract

The degree to which people take advice, and the factors that influence advice-taking, are of broad interest to laypersons, professionals, and policy-makers. This meta-analysis on 346 effect sizes from 129 independent datasets (*N* = 17, 296) assessed the weight of advice in the judge-advisor system paradigm, as well as the influence of sample and task characteristics. Information about the advisor(s) that is suggestive of advice quality was the only unique predictor of the overall pooled weight of advice. Individuals adjusted estimates by 32%, 37%, and 48% in response to advisors described in ways that suggest low, neutral, or high quality advice, respectively. This indicates that the benefits of compromise and averaging may be lost if accurate advice is perceived to be low quality, or too much weight is given to inaccurate advice that is perceived to be high quality. When examining the three levels of perceived quality separately, advice-taking was greater for subjective and uncertain estimates, relative to objective estimates, when information about the advisor was neutral in terms of advice quality. Sample characteristics had no effect on advice-taking, thus providing no evidence that age, gender, or individualism influence the weight of advice. The findings contribute to current theoretical debates and provide direction for future research.

The definition and measurement of advice varies across disciplines and research paradigms (MacGeorge & Van Swol, 2018a). It has been suggested that advice may be a higher-order factor subsuming lower-order factors, including, but not limited to, provision for or against a specific recommendation, and provision of guidance on how to make a decision (Bonaccio & Dalal, 2006). The current meta-analysis synthesises studies using the judge–advisor system (JAS) paradigm (Sniezek & Buckley, 1995), which is the most commonly applied measure of advice-taking. In this paradigm, the judge is asked to provide a numerical estimate (e.g., distance between two cities) before receiving an advisor’s (or advisors’) estimate(s). Then the judge is invited to revise their estimate, and sometimes an incentive is provided for accuracy. This allows for the calculation of the *weight of advice* using the formula: [(final estimate—initial estimate) / (advice – initial estimate)], which provides a continuous outcome on a scale from 0 (completely ignoring advice) to 1 (completely relying on advice) (Harvey & Fischer, 1997; Yaniv, 2004a). In the JAS, advice is broadly defined as information from another person, or people, or even an algorithm, that does not require advocacy by the advice-giver (Rader et al., 2017).

Receiving advice in the form of a numerical estimate that can be used to update an independent estimate represents one of the simplest forms of advice-taking and is ubiquitous in diverse real-world contexts. Professionals, such as physicians, weather forecasters, and financial advisors, as well as non-professional friends, family, and strangers, regularly provide advice in the form of quantitative estimates (e.g., number of calories in a meal, the chance of rain, cost of an investment or holiday, or online reputation ratings). The degree to which people incorporate advice into their decision-making has critical implications for public policy, including via vaccine refusal and climate change scepticism.

The average of more than one quantitative estimate usually results in a more accurate estimate if the advice is well-intentioned, and each estimate is independent of the other(s). Incorporating 50% of an advised estimate into an independent estimate would represent a rational use of advice (Larrick & Soll, 2006). However, the mean level of adjustment towards advice is typically around 30% (Soll & Larrick, 2009). This tendency can be explained by the theoretical construct of egocentric discounting whereby individuals weigh their own estimation more strongly than the estimations of others (Yaniv & Kleinberger, 2000). Previous narrative reviews that have focused on advice-taking (MacGeorge & Van Swol, 2018a), or social information use across five different tasks (Morin et al., 2021), or the JAS task specifically (Bonaccio & Dalal, 2006; Rader et al., 2017; Van Swol et al., 2018), concur that egocentric discounting is generally suboptimal yet poorly understood. Each of the three reviews that focused on the JAS task described the influence of multiple variables on egocentric discounting, while Bonaccio and Dalal (2006) stated that “once a critical mass of studies pertaining to each of these variables comes into existence, meta-analytic investigations will be in order.” (p. 139) A quantitative meta-analysis is useful for determining which variables may best explain egocentric discounting.

## Input-process-output model

**Fig. 1:**
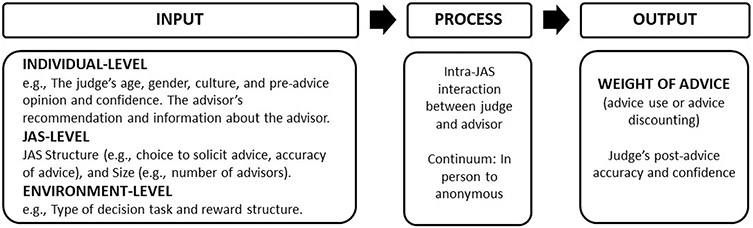
Judge-Advisor System Input-Process-Output Conceptual Framework

The conceptual framework for the current meta-analysis is based on the Bonaccio and Dalal (2006) input-process-output (IPO) model for explaining weight of advice in the JAS (see Fig. 1). The “input” category in the IPO model comprises of individual-level, JAS-level, and environment-level factors. Individual-level factors include the judge’s pre-advice opinion and confidence, as well as information about the advisor. JAS-level factors include whether advice is optional or imposed, and the number of advisors. Environment-level factors include the type of decision task and reward structure (e.g., whether financial incentives are available and whether they are tied to decision accuracy). The “process” category accounts for intra-JAS interaction between the judge and advisor(s) on a continuum from in-person to partially concealed to completely anonymous. The “process” category and the “input” factors both predict the “output” which includes the weight of advice (advice use or discounting), as well as the judge’s post-advice accuracy and confidence. The present meta-analysis used the JAS IPO model (Bonaccio & Dalal, 2006) as a framework to examine the influence of “input” factors on weight of advice, and in particular the influence on advice discounting. The “process” category was not assessed because most studies involved anonymous interactions.

## Input factors

Following data extraction, the most commonly measured variables included the judge’s age, gender, and culture (i.e., degree of individualism), as well as the perceived advice quality based on information about the advisor, and the type of decision task (i.e., objective and certain versus subjective and uncertain).

### Judge age

The weight given to advice may decrease from early childhood through adolescence (Molleman et al., 2021; Rakoczy et al., 2015), and then increase again after the age of 65 years (Bailey et al., 2021a). Dual process models of ageing and decision-making suggest reduced deliberation with age and therefore a motivational shift away from autonomous decision-making in young adulthood and towards increased joint decision-making and reliance on others (Peters et al., 2007). There is also evidence that advice-taking is positively associated with trust (Bonaccio & Dalal, 2006), and trust increases with age (Bailey et al., 2021b), further suggesting greater weight of advice as age increases. An analysis of the influence of age between the ages of 18 and 65 years would provide a broader view of the trajectory of advice-taking across the adult lifespan.

### Judge gender

Trust also differs as a function of gender. One study showed that men are more trusting than women in the economic trust game and suggested that men viewed the interaction more strategically than women (Buchan et al., 2008). However, a meta-analysis provided evidence that women are more trusting than men (Feingold, 1994), and co-workers have been shown to report that women take more advice than men (See et al., 2011). In contrast, MacGeorge et al. (2016) found that gender has only limited influence on advice evaluation, albeit in the context of discussing a problem with a friend. Nevertheless, the confidence literature further suggests that there may be increased advice-taking among women relative to men. Confidence is negatively associated with advice-taking (Rader et al., 2017), and women are less confident than men when making judgments (See et al., 2011). The current meta-analysis presents an opportunity to establish a clearer picture of the potential effect of gender on advice-taking in the judge-advisor task. This can be achieved by assessing the influence of the proportion of female judges in each sample on the weight of advice.

### Judge culture

Individualist versus collectivist cultures may differ in the degree to which they integrate advice from others into judgment and decision-making. Members of collectivist cultures desire relational harmony, obey authority, and are more likely to perceive and understand advice from others (Tinghu et al., 2018). This suggests increased advice-taking among cultures that are more collectivistic. In contrast, Gheorghiu et al. (2009) found that individualism was more likely to foster trust among people than collectivism, and as previously discussed, trust may increase advice-taking. They explained this finding in light of Yamagishi and Yamagishi’s theory that norms emphasising independence, autonomy, and distinctiveness, which are characteristic of individualistic cultures, are more likely to foster trust among people (Yamagishi et al., 1998). Alternatively, individualists may discount advice because it undermines their desire for autonomy and motivation to maintain a favourable self-concept (Rader et al., 2017). The Morin et al. (2021) narrative review concurs that there is mixed empirical evidence for the effect of culture on advice-taking. A quantitative synthesis of the existing data will help to disentangle this evidence to determine whether there is a systematic effect of individualism on advice-taking.

### Perceived advice quality

JAS studies to date have manipulated participants’ perceptions of advisors, and therefore perceptions of advice quality, in a number of ways. They have explicitly referred to advisors as novices or experts with low versus high expertise, respectively (e.g., Bailey et al., 2021a; Meshi et al., 2012). Alternatively, advisors have been described in neutral terms, as other participants, supposedly similar to the current participant (e.g., Gino, 2008; See et al., 2011). Advice has also been described as the average of estimates provided by a number of previous participants, suggesting high quality (i.e., Carbonell et al., 2019; Logg et al., 2019). Greater activity in the ventral striatum, a brain region associated with the anticipation of reward (Knutson et al., 2001), was identified when participants were told to expect expert rather than novice advice (Meshi et al., 2012). This suggests that expert advice is valued more highly than novice advice, perhaps due to an expectation of it being high quality and leading to an improvement in performance. We included descriptions of the advisor that suggest advice quality as a task characteristic that was not previously specified as an input factor in the JAS IPO model.

The informational asymmetry account suggests that egocentric discounting occurs because people have greater access to their own reasons for a judgment relative to the reasoning behind another person’s judgment (Yaniv, 2004b; although see Trouche et al., 2018). This assumption is supported by evidence for increased advice-taking when self-reported knowledge is low (Duan et al., 2021; Yaniv & Choshen-Hillel, 2012) or the decision is difficult (Gino & Moore, 2007). Having more information about the advisor, such as about their level of expertise, is likely to reduce informational asymmetry. Indeed, Yaniv and Kleinberger (2000) suggest that a judge will form a view of the advisor following repeated interactions, and that this reputation formation will influence the weight of advice. They further argue that risk aversion contributes to asymmetry of reputation formation because the risk of an average advisor giving bad advice looms larger than the benefit of receiving good advice. This means that average and bad advisors would be considered similarly, despite average advisors sometimes giving good advice. Monitoring of behaviour across repeated interactions is not always possible and consequently, reputation is often derived from available information in one-off interactions or via second-hand information. The latter is the most common method for conveying the quality of the advisor in the JAS paradigm, and the degree to which reputation asymmetry influences advice-taking from advisors likely to provide low, average, or high quality advice remains to be established.

### Uncertainty of estimate

The uncertainty of the estimate may influence advice-taking but has received little attention in the literature to date. Objective values have one correct answer (e.g., the number of coins in a jar or distance between two cities). However, for more subjective or uncertain judgements (e.g., stock forecasting or service-provider ratings), there is not necessarily a single correct answer. Subjective or uncertain judgments, which are based on opinion, may result in greater advice-taking than objective judgments because ultimately their correctness is determined by a consensus among individuals (Laughlin & Ellis, 1986). Indeed, uncertainty about an initial estimate is a good predictor of advice-taking (Gino, 2008). Uncertainty also implies low knowledge, which may increase the perception that the advisor is more knowledgeable than the decision-maker and therefore increase advice-taking (Gino & Moore, 2007; Yaniv & Kleinberger, 2000; Yaniv, 2004a, b). An alternative proposition is that advice will be given more weight when estimating objective relative to subjective values. A primary motivation for taking advice is to improve accuracy (Rader et al., 2017). This suggests that because subjective estimates do not have a right or wrong answer, advice offers less opportunity to improve accuracy relative to advice relating to an objective estimate (See et al., 2011). Indeed, Van Swol (2011) demonstrated that decision-makers take more advice when determining a cognitively challenging objective estimate relative to a subjective estimate based on personal taste. The current meta-analysis will test these competing theoretical arguments for disproportionate use of advice when estimating subjective and uncertain versus objective and certain values.

## The current meta-analysis

We build upon existing narrative reviews (i.e., Bonaccio & Dalal, 2006; MacGeorge & Van Swol, 2018a; Morin et al., 2020; Rader et al., 2017; Van Swol et al., 2018) and the IPO conceptual framework to conduct a systematic review and meta-analysis of advice-taking in the JAS task. In their review, Bonaccio and Dalal (2006) pointed to the need for research to extend an analysis of situational variables that influence advice-taking, to also include individual difference variables. Thus, we sought to provide the first synthesis of data to examine whether advice-taking is influenced by age, gender, or cultural context. We also intended to contribute to competing theoretical arguments relating to the potential influences of gender, culture, and estimate subjectivity on weight of advice. Morin et al.’s (2020) review identified mixed evidence for the effect of culture in advice-taking. Further, culture operationalised as a degree of individualism has the potential to contribute to an understanding of the roles of self-concept and desire for autonomy, which Rader et al.’s (2017) narrative review highlighted as motives that may be particularly important in advice-taking. The previous reviews did not address estimate subjectivity in any depth and as such the current meta-analysis will provide an initial review of this potential influence on advice-taking.

A further aim was to establish the influence of perceptions of the advisor on advice-taking, and particularly whether decision-makers differentiate advisors perceived to provide low or neutral quality advice. Based on Yaniv and Kleinberger’s (2000) conceptual framework for understanding advice-taking, we expected that information about the advisor indicating advice quality would be the strongest influence on the overall mean weight of advice. We therefore planned further analyses to separately examine predictors of the weight of advice in response to advisors described as providing (1) high, (2) neutral, and (3) low quality advice. In addition to examining mean age, the percentage of females, culture, uncertainty of estimate, and perceived advice quality as predictors of advice-taking, we extracted further potential predictors if they were amenable to meta-analysis. These included, the judge’s pre-advice confidence, type of sample (i.e., student vs non-student), actual advice accuracy, whether the judge was offered an accuracy incentive, type of incentive for participating in the study (cash versus course credit), whether advice was imposed versus optional, and number of advisors (single versus multiple).

## Method

This study was conducted according to the Preferred Reporting Items for Systematic Reviews and Meta-Analyses (PRISMA) guidelines (Moher et al., 2009). Anonymised data and code are accessible at the Open Science Framework (https://osf.io/atz6y/?view_only=a5e435f0b5de42a286736725a11bb58d).

### Information sources and search

**Fig. 2:**
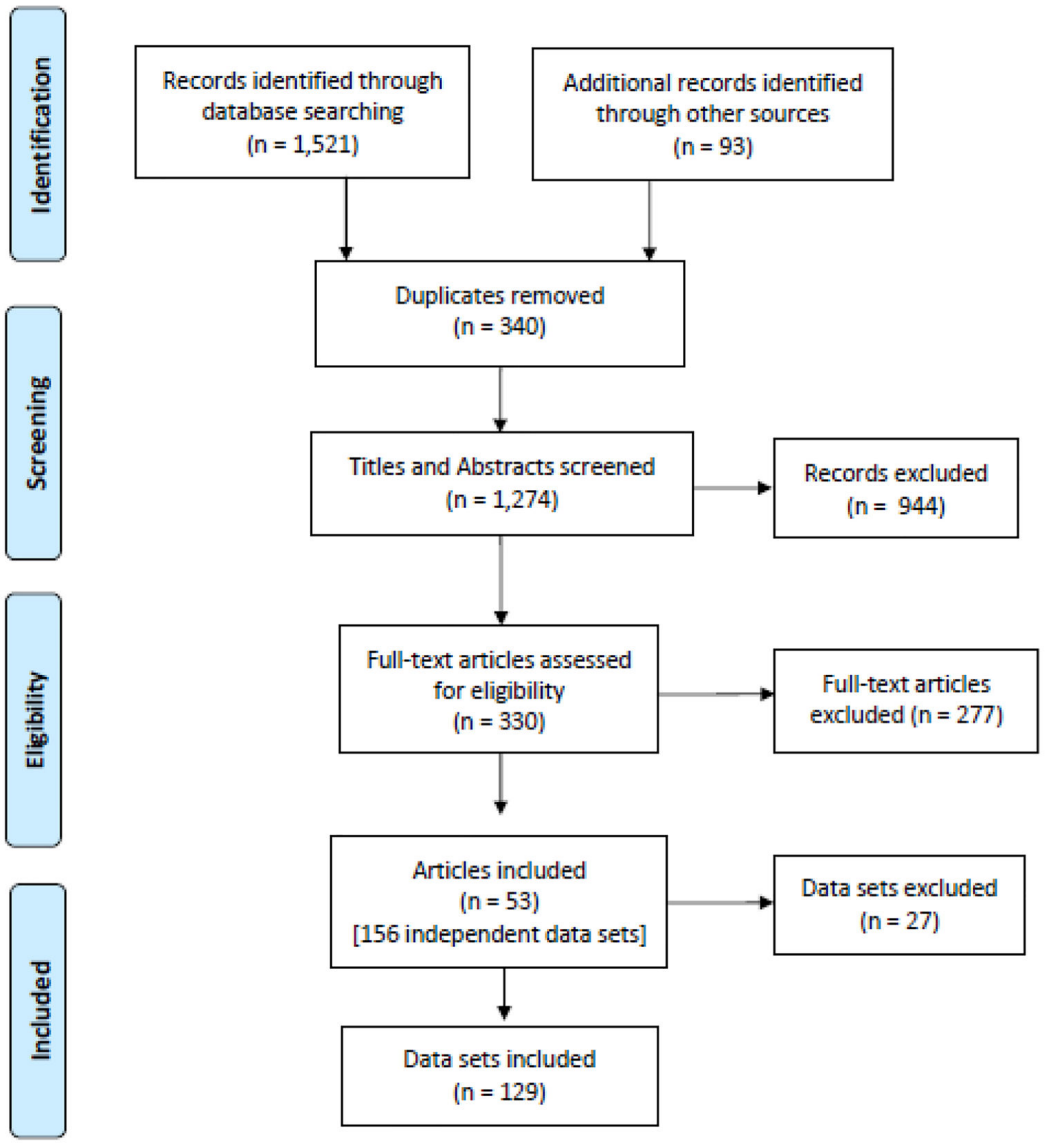
PRISMA Flowchart Depicting Selection of Studies for Meta-Analysis on Advice-Taking

A computerised literature search using PsycINFO, PubMed, Web of Science, and Scopus was completed on 21 February, 2020. The search did not apply any limitation on the year of publication. The title, keyword, and abstract search terms included: “use of advice” OR “advice use” OR “advice seeking” OR “advice taking” OR “weight of advice” OR “judge-advisor system” OR “judge-adviser system” OR “judge-advisor” OR “judge-adviser” OR “advice utilization” OR “advice utilisation”. In December 2020 we emailed the corresponding author from each identified paper that was published within the past 10 years to request unpublished data. At the same time, we posted a call for unpublished data on the Society for Judgment and Decision Making mailing list, and performed a search of preprints in OSF. Manual forward (review of articles that were cited in the final set of articles) and backward (review of articles that were cited in the final set of articles) citation searches were conducted February 2021. A PRISMA flowchart outlines the process for selecting studies for inclusion in this meta-analysis (see Fig. 2).

### Eligibility criteria

Studies were included if (1) the paper was written in English, (2) advice-taking was measured using the JAS paradigm, (3) weight of advice was calculated using the formula: [(final estimate—initial estimate) / (advice – initial estimate)] or a variant of this formula, such as [final estimate—initial estimate / advice – initial estimate], and (4) statistics for calculating effect size were available in the paper or supplied by the author.

### Data extraction and study selection

Table 1 reports the sample and task characteristics for each independent data set. Authors PB and TL extracted all data. When data were not available in tables or text, but figures were available, we used WEBPLOTDIGITIZER software to extract the data from the figures. Estimating data in this way has been shown to involve a small margin of error but to be “satisfactory, accurate, and efficient” (Burda et al., 2017, p. 260). If no form of data was available in a paper, we contacted the corresponding and/or first author via email. Two attempts were made to contact authors and effects were excluded when we received no response or were informed that data were no longer available (i.e., Sciandra, 2019; Scopelliti et al., 2015; See et al., 2011; Study 4; Sniezek et al., 2004; Tzioti et al., 2014; Yaniv, 2004a; Yaniv & Kleinberger, 2000, Studies 2 to 4; Wanzel et al., 2017).

PB extracted data for each effect size (*M*, *N,* and *SD* or *SE*) a second time to ensure 100% reliability across the two independent data files. When only the total number of participants was available across multiple conditions, we assumed even numbers of participants in each condition. Data extracted using WebPlotDigitizer often differed by decimal places across the two extractions, and, in such cases, we used the average of the two extractions. PB extracted the predictor data from the included studies. TL checked the extracted predictor data for errors, and disagreements were resolved by discussion and consensus. An independent coder then extracted predictor data for 20% of the 129 studies (i.e., 26 studies). There was initially 96% agreement between the independent coder and the coding completed by the authors. The inspection of discrepancies revealed errors in 6.3% of the independent coder’s extractions. Removing these errors there was 99% agreement between coders.

Twelve effects that reflected group rather than individual decision-making were excluded (Kim et al., 2020; Larson et al., 2020), as were eleven effects based on the decisions of dyads (Minson & Mueller, 2012; Schultze et al., 2019). Four effects were excluded because participants were presented with manipulated initial estimates at the same time that they were provided with advice (Trouche et al., 2018). A summary of the predictor names, definitions, operationalisations, and representative sources is provided in Table 2. In-depth explanations of predictor coding decisions are provided as Supplementary Information.

### Meta-analytic approach

This meta-analysis of proportion data synthesises a one-dimensional binomial measure known as the (weighted or pooled) average proportion. This is the average of proportions within multiple studies weighted by the inverse of their sampling variances. Raw proportion of advice-taking was used as the effect size index because observed proportions were around 0.5 and the number of studies was sufficiently large (Barendregt et al., 2013), and also because a re-analysis of the data using a logit transformation did not change the significance of any finding. A larger proportion indicates a greater degree of adjustment of an estimate *towards* the estimate of an advisor or advisors.

Dependency refers to violation of the statistical assumption that effect sizes are independent. One type of dependency in meta-analysis arises from individual studies contributing multiple effect sizes. We dealt with dependency of effects within studies by following the steps described in Assink and Wibbelink (2016) for fitting a three-level meta-analytic model using the metafor package (Viechtbauer, 2010) in R (Version 4.1.2; R Core Team, 2021). Variance components are distributed over three levels of the model: individual level sampling variance (level 1); variance between effect sizes within studies (level 2), and variance between studies (level 3), as described by Van den Noortgate et al. (2013). Parameters were estimated using the restricted maximum likelihood procedure. An ANOVA function tested the fit of a three-level model against the two-level models. weight of advice is measured as a proportion (ranging from 0 to 1). To examine potential predictors of the overall effect in each three-level model, continuous variables were centered around the variable mean and were assessed using a three-level meta-regression model. Categorical predictors with *k* categories were converted to k-1 dummy variables through binary coding and were assessed using a three-level mixed-effects model. Testing multiple significant predictors in a single model after potential effects have been evaluated separately in univariate models is a reasonable strategy for dealing with potential multicollinearity (Hox, 2010). Variance inflation factors (VIFs) were also calculated to test for multicollinearity.

It is not possible to test for publication bias using trim-and-fill or Egger’s test in a multilevel meta-analysis. We therefore tested for publication bias using one pooled estimate of the weight of advice for each study. When an individual study included two or more conditions (i.e., dependent outcomes), effect sizes for each outcome were pooled. We used the MAd package (Del Re & Hoyt, 2014) in R to create the composite estimate using recommended procedures as described in The Handbook of Research Synthesis and Meta-Analysis (Cooper et al., 2009). The composite was calculated accounting for a conservative correlation of 1.0 among within-study outcomes and implemented the Borenstein et al. (2009) procedures for aggregating dependent effect sizes.

After imputing missing studies to form a symmetrical funnel plot, the trim-and-fill method provides an estimate of the true mean and variance (Duval & Tweedie, 2000). Egger’s test assesses the degree of asymmetry in the funnel plot as measured by the intercept from regression of standard normal deviates against precision (Egger et al., 1997).

## Results

### Study selection and characteristics

As summarised in Fig. 2, the initial literature search resulted in 355 articles in PsycINFO, 158 articles in PubMed, 453 articles in Web of Science, and 555 articles in Scopus (*n* = 1,521). After merging the four databases, 340 duplicates were removed. An additional 93 articles were identified using other methods described in Information Sources and Search. 944 records were excluded following the screening of the titles and abstracts, and a further 277 following screening of the full paper. The final data consisted of 53 articles comprising 129 independent data sets with a total of 17,296 participants. From these data sets we extracted 346 effect sizes.

### Overall pooled effect

**Fig. 3:**
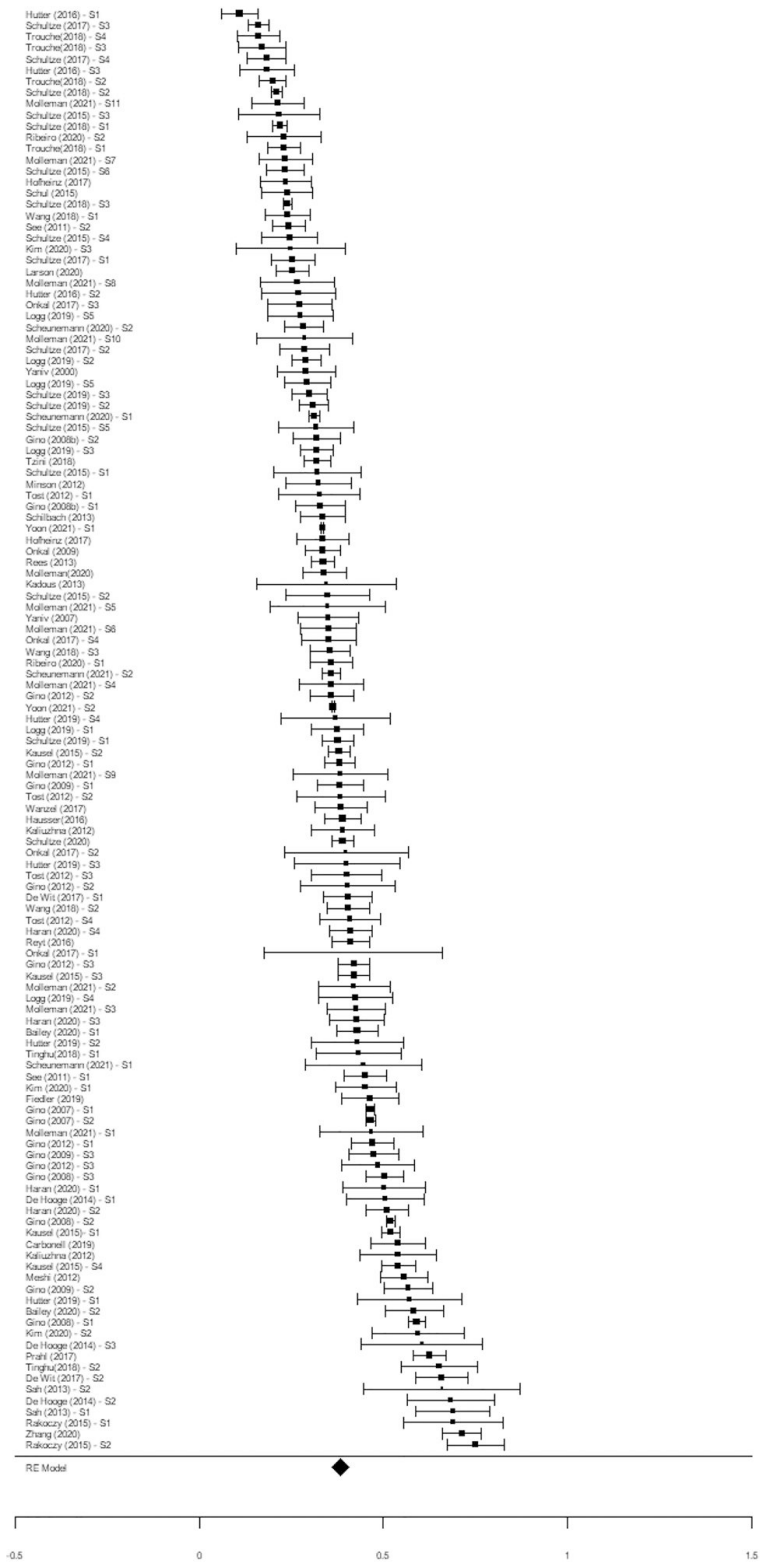
Forest Plot of the Overall Weight of Advice. *Note*. The diamond represents the overall pooled weight of advice proportion. Each effect size and 95% confidence interval (error bar) represents an independent sample (*s* = 129). For articles with multiple independent samples, the effect size for each sample (S1, S2, etc.) is reported separately. Where a sample contributed more than one effect, the pooled effect, accounting for dependency between effects, is represented

We conducted multi-level meta-analysis using the rma.mv function of the ‘metafor’ package (Viechtbauer, 2010). Our three-level meta-analytic model showed that the overall pooled weight of advice (*k* = 346) was 0.39, 95% CI [0.37, 0.42]. This overall effect was significant, *t*(345) = 31.57, *p* < 0.001, and indicates that individuals, on average, adjusted their estimates to be 39% closer to an advised estimate/s (see Fig. 3). A boxplot identified two outlier effect sizes (0.93 and 0.92). Exclusion of these two data points did not substantially change the overall effect, 0.39, 95% CI [0.37, 0.42], *t*(343) = 31.97, *p* < 0.001, and so they were retained in subsequent analyses.

We compared the fit of the original three-level model with the fit of a two-level model in which within-study variance (level 2) was not modelled. We found that the fit of the original three-level model was statistically better than the fit of the two-level model (*p* < 0.0001), suggesting that there was significant heterogeneity between effect sizes within studies. Next, we compared the fit of the original three-level model to the fit of a model where only variance at level 2 was freely estimated and where the variance at level 3 (between-studies), was fixed at zero. We found that the fit of the original three-level model was statistically better than the fit of the two-level model (*p* < 0.0001), suggesting that there was significant heterogeneity between studies. The estimated variance components between effect sizes within- and between-studies were *τ*^2^_Level2_ = 0.012 and *τ*^2^_Level3_ = 0.015, respectively. Of the total variance, 0.16 percent was attributed to variance at level 1 (i.e., sampling variance); 57.08 percent was attributed to differences between effect sizes within samples at level 2 (i.e., within-study variance); and 42.76 percent was attributed to differences between studies at level 3. We therefore extended our model to examine the potential influence of additional variables.

### Multiple predictor model

An analysis with multiple predictors was conducted to examine the unique influence of each significant univariate model predictor (perceived advice quality, estimate uncertainty, and accuracy incentive) on the summary weight of advice (see Supplementary Information for the univariate models). We excluded actual advice accuracy because only 35% of effect sizes could be coded for accuracy. There was no evidence of multicollinearity among the predictor variables as evidenced by VIFs ≤ 1.36. The overall model was significant, *F*(4,279) = 10.82, *p* < 0.001. Only high, *t*(279) = 3.70, *p* < 0.001, and low, *t*(279) = 2.10, *p* = 0.037, perceived advice quality (relative to neutral perceived advice quality) had unique effects not confounded by other variables in the model.

### Advice-taking as a function of perceived advice quality

Next, we separately examined the weight of advice in response to advisors perceived to be providing (1) high quality advice, (2) neutral quality advice, and (3) low quality advice. Because there were no predictors of advice-taking when advice was perceived to be either high or low quality, we report these data in the Supplementary Information.

#### Advice perceived as neutral quality

**Fig. 4:**
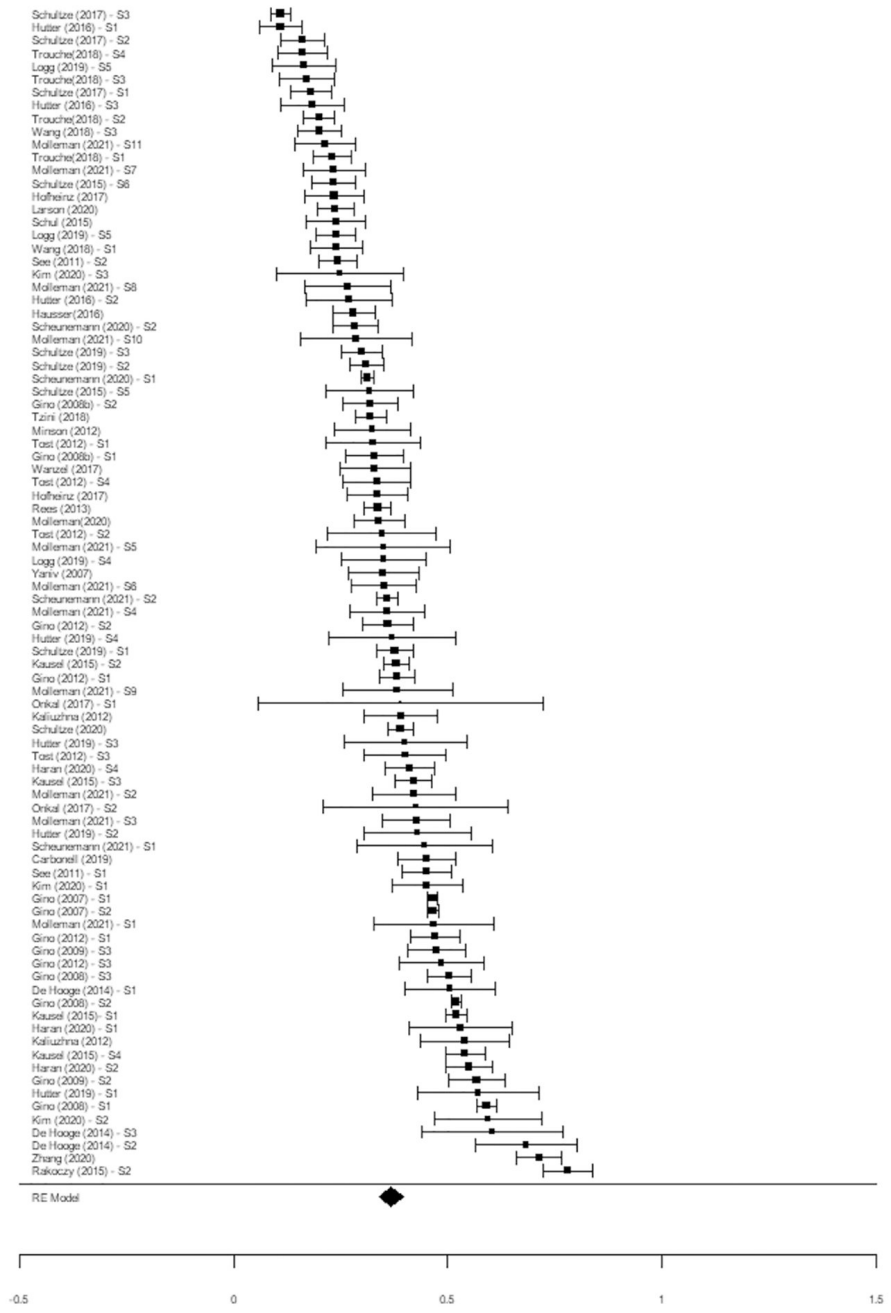
Forest Plot of the Weight of Advice in Response to Advisors Perceived to Provide Neutral Quality Advice. *Note*. The diamond represents the summary pooled weight of advice proportion. Each effect size and 95% confidence interval (error bar) represents an independent sample (*s* = 90). For articles with multiple independent samples, the effect size for each sample (S1, S2, etc.) is reported separately. Where a sample contributed more than one effect, the pooled effect, accounting for dependency between effects, is represented

The summary effect when the advisor was perceived to provide neutral quality advice (*k* = 170) equaled 0.38, 95% CI [0.35, 0.40], *t*(169) = 25.46, *p* < 0.001 (see Fig. 4). A boxplot identified no outlier effect sizes.

The original three-level model was a better fit than the two-level model in which level 2 (within-study variance) was not modelled (*p* < 0.0001), as well as the two-level model where level 3 (between-study variance) was fixed at zero (*p* < 0.0001). Consequently, there was significant variability between effect sizes within- and between-studies, and the estimated variance components were *τ*^2^_Level2_ = 0.009 and *τ*^2^_Level3_ = 0.015, respectively. Of the total variance, 1.45 percent was attributed to variance at level 1 (i.e., sampling variance); 61.95 percent was attributed to level 2 (i.e., within-study variance); and 36.59 percent was attributed to level 3 (i.e., between-study variance). We therefore extended our model to examine potential predictors.

#### Multiple predictor model

An analysis with multiple predictors was conducted to examine the unique influence of each significant univariate model predictor (estimate uncertainty, accuracy incentive, and participation payment) on the summary weight of advice (see Supplementary Information for the univariate models). There was no evidence of multicollinearity among the predictor variables as evidenced by VIFs ≤ 1.32. The overall model was significant, *F*(3, 124) = 8.93, *p* < 0.001. Only estimate uncertainty, *t*(124) = 4.10, *p* < 0.001, had a unique effect that was not confounded by other variables in the model.

### Publication bias and power

**Fig. 5:**
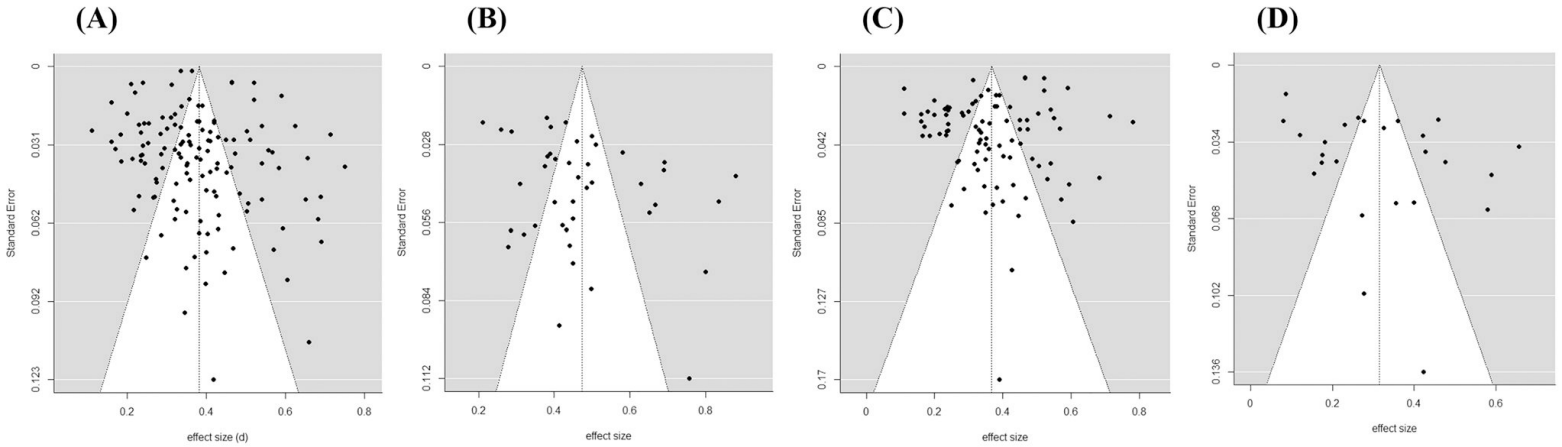
Funnel Plots for Studies Examining the Weight of Advice **(A)** Overall (s = 129), or in Response to Advisors Perceived to Provide **(B)** High (s = 45), **(C)** Neutral (s = 90), and **(D)** Low (s = 24) Quality Advice. *Note*. *s* = number of independent data sets contributing to the analysis

To determine whether there was evidence of publication bias, we first visually inspected a funnel plot displaying the aggregated within-study effect size estimates and standard errors (see Fig. 5). A pattern of asymmetry in the funnel plot suggests potential publication bias. The Trim and Fill method imputed eight missing studies to the left of the mean overall effect, and Egger’s regression test detected significant bias (*p* = 0.012). We therefore cannot rule out publication bias.

Next, we inspected funnel plots for displaying the aggregated within-study effect size estimates and standard errors separately for advice-taking in response to advisors perceived to provided high, neutral, and low quality advice (see Fig. 5). The Trim and Fill method imputed eight missing studies to the left of the mean effect for advisors perceived to provide high quality advice, and the updated estimate of the pooled effect size was 0.41, 95% CI [0.36, 0.47], but Egger’s regression test detected no significant bias (*p* = 0.122). Two missing studies were imputed to the left of the mean effect for advisors perceived to provide neutral advice quality, and the updated estimate of the pooled effect size was 0.36, 95% CI [0.33, 0.39], but Egger’s regression test detected no significant bias (*p* = 0.139). Two missing studies were imputed to the left of the mean effect for advisors perceived to provide low quality advice, and the updated estimate of the pooled effect size was 0.29, 95% CI [0.22, 0.36], but Egger’s regression test detected no significant bias (*p* = 0.194).

Power analysis shows that we had 100% power to detect a small overall effect (*d* = 0.2) based on *k* = 346 and an average sample size of 134, regardless of the degree of heterogeneity (Valentine et al., 2010). If *k* = 38, as for studies that include advice from advisors perceived to provide low quality advice, we had 99.9% power to detect a small overall effect (*d* = 0.2) with the same average sample size. Power increases as the number of studies (*s*) and effect sizes (*k*) increase (Assink & Wibbelink, 2016). To ensure sufficient power, meta-regression requires at least 10 studies per predictor (Higgins & Green, 2006). We met this threshold for the two multiple predictor models that included three predictors each (*k* = 284, *s* = 117; *k* = 128, *s* = 67). We also met this threshold for the univariate model with the smallest number of studies (*k* = 31, *s* = 18). Nevertheless, any null effects should be interpreted with caution.

## Discussion

The current meta-analysis examined the extent to which individuals use advice, as well as predictors of this behaviour. The combined results from 346 effect sizes within 129 independent data sets from 53 articles suggest that, on average, estimates are adjusted 39% towards advised estimates. This is less than the 50% that is considered a rational adjustment towards the estimate of an advisor, based on the statistical principle that aggregation of imperfect estimates reduces error (Larrick & Soll, 2006). Publication bias analyses showed that this tendency towards egocentric discounting of advice may be even stronger than suggested in the literature to date. Our analyses also revealed that characteristics of the sample do not predict the weight of advice, providing no evidence that advice-taking is influenced by age, gender, or individualism. The most significant predictor of advice-taking was information about the advisor suggesting the potential quality of the advice. When information about the advisor(s) was unavailable or neutral, more weight was given to advice when the estimation was based on a subjective or uncertain value compared to an objective or certain value.

### Individual-level predictors of advice-taking

#### Characteristics of the judge

Although this meta-analysis did not support an effect of age on advice-taking, only two studies involved participants younger than 18 years of age (Molleman et al., 2021; Rakoczy et al., 2015), and only one study involved older adults (aged 65 years or more; Bailey et al., 2021a). We therefore cannot rule out maturation and socialisation influencing advice-taking prior to reaching young adulthood or in older age. However, these processes do not appear to influence advice-taking throughout adulthood. Similarly, there was no influence of degree of individualism on advice-taking. A suggestion that remains to be tested in future research is that geographical differences, including economic and psychosocial adversity, may have more of an influence on advice-taking than culturally transmitted ideologies (Morin et al., 2020). Alternatively, motivations underlying advice-taking, rather than degree of advice-taking, may differ for individualistic and collectivistic cultures. Whereas increased advice-taking in individualistic cultures may be motivated by the desire for autonomy and maintenance of self-concept (Rader et al., 2017), collectivistic cultures may be motivated by relational harmony, even in anonymous, one-off JAS interactions (Tinghu et al., 2018). Future studies should examine whether additional cultural differences such as power distance and advice-giver authority, and their interaction, influence advice-taking.

Increased trust and reduced confidence are associated with both greater advice-taking (Bonaccio & Dalal, 2006; Rader et al., 2017) and being female (Feingold, 1994; See et al., 2011). However, gender (i.e., percent female; 0.04% to 81.33% of each sample) was not a predictor of the weight of advice. There is some evidence that the effect of gender on trust may depend on the type of trust. For example, men have been found more trusting than women in an economic trust game when financial incentives are present (Buchan et al., 2008). Differing incentives between studies may have influenced trust-based gender effects in the JAS paradigm. Similarly, the effect of gender on confidence and therefore advice-taking may depend on context. Previous research showing that women are less confident in their judgements and take more advice than men was in the context of existing co-worker relationships (See et al., 2011). In contrast, the JAS paradigm typically involves one-off, anonymous interactions. Nevertheless, the current data may simply reflect a lack of any effect of gender on advice-taking.

#### Characteristics of the advisor

A mean weighting of 48% was evident in response to advice from advisors perceived to provide high quality advice. This is closely approaching Larrick and Soll’s (2006) suggested rational weighting of 50%, and suggests that egocentric discounting may not be as pervasive as suggested in the literature to date. It may also be argued that a rational weighting should be greater than 50% if the advisor is perceived to be providing high quality advice. Given people consider experts to provide more influential and helpful, and less intrusive advice (Dalal and Bonaccio, 2010), it is not surprising that greater weight is given to advisors perceived to be providing high quality advice, including those described as experts. Critically, however, only *perceptions* of the accuracy of the advisor, and not actual advice accuracy or knowledge of actual accuracy, uniquely predicted advice-taking.

The mean weight of advice in response to advisors perceived to provide low quality advice (i.e., 32%) did not differ from the degree of advice-taking from advisors who were described in neutral terms (i.e., 37%). This is consistent with asymmetry of reputation formation over repeated interactions, which in turn is explained by risk aversion theories (Yaniv & Kleinberger, 2000). Specifically, the risk of an average advisor giving bad advice looms larger than the possibility that the advisor may provide good advice. We extend evidence for this effect from repeated interactions that involve progressive learning to one-off interactions and repeated interactions that do not involve feedback. An important distinction between these different methods of reputation formation is that first impressions are not always reliable. Thus, without first-hand evidence of the quality of advice, there is a risk that too much weight is given to advice from an unreliable advisor, or too little weight to good advice from an unknown advisor.

#### JAS-level and environment-level predictors of advice-taking

The current data contribute to clarification of competing theoretical propositions regarding the influence of estimate uncertainty on advice-taking. When information about the advisor is lacking, objective estimates are adjusted by 35%, while subjective/uncertain estimates are adjusted 55% towards advice. This greater weight of advice when determining a subjective estimate may reflect an understanding that subjective values are typically determined by aggregation (Laughlin & Ellis, 1986). It may also suggest that the judge perceives that their own knowledge of the estimate is uncertain and potentially reduced relative to the knowledge of the advisor, and this in turn may increase advice-taking (Gino & Moore, 2007; Yaniv & Kleinberger, 2000; Yaniv, 2004a, b). This type of knowledge comparison may occur more frequently when judges do not have information about the advisor that suggests the potential quality of the advice. Given previous evidence for a negative association between confidence and advice-taking (Bonaccio & Dalal, 2006), it is also possible that an uncertain estimate reduces the judge’s confidence which in turn increases advice-taking.

We did not find evidence for the alternative proposition that advice would be given more weight when estimating objective relative to subjective values because only the former offers the opportunity to improve accuracy. A preference for advice in relation to a subjective estimate where there is no single correct answer may suggest that the JAS is not always dominated by accuracy-seeking informational motives (See et al., 2011; Van Swol, 2011), but may also assess normative social influence and the motivation to maintain social harmony (Mahmoodi et al., 2015). Rader et al.’s (2017) review identified a focus on informational motives as both a strength and a limitation of the existing JAS literature. They recommended that future research reconnect with the social influence literature and normative motives within the JAS task. Our data suggest that these motives contribute to understanding egocentric discounting, and that future JAS research should examine the role of normative motives in reducing suboptimal egocentric discounting.

Although accuracy incentives and advice accuracy were predictors of advice-taking in the univariate models, they were not unique predictors of the mean weight of advice. There was also no influence of whether advice was imposed versus optional, or for multiple pieces of advice versus a single piece of advice. It should be noted that few studies included in the current meta-analysis examined whether advice was optional (< 5%) or the influence of receiving multiple pieces of advice (< 6%). Nevertheless, we considered these variables important to analyse given that they are input factors in Bonaccio and Dalal’s (2006) JAS IPO model.

### Limitations and future directions

The current meta-analysis was the first to quantify the magnitude of advice-taking and the variables that influence this behaviour. We extended Bonaccio and Dalal’s (2006) input-process-output model to include perceptions of the advisor as a specific input factor that may predict advice-taking. We further broadened the focus of this model on situational influences as inputs (i.e., task characteristics) to include individual difference variables (i.e., decision-maker characteristics). The analysis is not without limitations, which are largely a consequence of the existing data sets. For example, additional characteristics of the advisor are likely to influence advice-taking. This includes trustworthiness, likability, and similarity to the judge (Feng & MacGeorge, 2010; MacGeorge & Van Swol, 2018b). These characteristics are not commonly measured in studies using the JAS paradigm. Likewise, there are several sample characteristics which were not analysed and which may nonetheless have an effect on advice-taking. This includes, but is not limited to, the judge’s expertise, personality, or desire for autonomy. Only a few studies have provided data to allow for an examination of advisor confidence. This individual-level factor is likely to interact with other variables such as advisor accuracy (Sah et al., 2013) or estimate uncertainty (Van Swol, 2011).

It was also not possible to measure the process category of the IPO model as a predictor. In contrast to the input level, the “process” level in the JAS IPO model, involving intra-JAS interaction between the judge and advisor, or between multiple advisors, is relatively neglected. For example, 111 out of 129 samples in the current meta-analysis interacted with advisors via a computer. Two interacted via telephone (i.e., Gino & Schweitzer, 2008, Study 1; Gino et al., 2009, Study 1), two face-to-face (Tinghu et al., 2018, Study 1, Study 2), one via web-cam (De Wit et al., 2017, Study 3), and 12 in writing (e.g., Kaliuzhna et al., 2012). One study did not specify the form of interaction (Minson & Mueller, 2012). Most studies using the JAS paradigm have involved anonymous interactions between judge and advisor. To adequately assess the effects of the process level, the next step will be to measure real-life judge-advisor interactions using more naturalistic methods such as experience sampling.

One of the methodological difficulties with the weight of advice metric as currently determined is that it does not capture instances where estimates move *away* from advice. In the JAS paradigm, this is typically adjusted to zero. However, a score of zero indicates that advice was simply ignored, rather than caused the judge to move their estimate in the opposite direction. This ensures that the average weight of advice is always positive, which biases the results toward finding evidence for advice-taking. Generally, this is not a substantial problem as most adjusted estimates fall between the initial estimate and the advice and so are not adjusted to zero (Harvey & Fischer, 1997). However, future JAS research should address the difficulties with the classic weight of advice formula to capture circumstances where the judge may incorporate advice but in the opposite direction to that suggested by the advisor. Future studies should also explore advice-taking calculations that account for non-linear dynamics of the opinion aggregation process. The current meta-analysis focused on adjustment of quantitative estimates following advice and did not examine the effects of advice-taking for decisions that involve choosing between qualitatively different options. Nevertheless, the current approach addresses a common criticism of meta-analysis, which is the problem of mixing ‘apples and oranges’ (Sharpe, 1997).

## Conclusion

In conclusion, the most significant predictor of advice-taking was information about the advisor(s) that suggested low, neutral, or high quality advice. However, risk aversion effects appeared to diminish differentiation of advisors perceived to provide low quality or neutral quality advice. Taken together, the benefits of compromise and averaging may be lost if accurate advice is perceived to be low quality, or too much weight is given to inaccurate advice that is perceived to be high quality. When there was no information about the advisor with which to establish the potential quality of advice, advice-taking increased when the estimate was of a subjective or uncertain nature relative to when there was an objectively correct answer, suggesting that normative motives may increase JAS advice-taking. The current data provide no evidence that advice-taking is influenced by age, gender, or individualism, while noting there is relatively little data about the effects of more extreme age groups on advice-taking. These findings provide an important evidence base across diverse contexts, from policy-makers tasked with advising the public to reduce risks, to professionals such as doctors advising patients with health-related information, or friends and families passing on uninformed financial advice. An understanding of advice-taking is critical for ensuring optimal integration of social information into independent judgment.

## Supplementary Material

Supplementary Information

## Figures and Tables

**Table 1:** Sample Characteristics, Task Characteristics, and Pooled Weight of Advice (as a Proportion) for Each Independent Sample Included in the Meta-Analysis

	Sample characteristics						Task characteristics						
Study	*N*	Age (*M*)	Type	% female	Country	Individualism	Participation payment	Uncertain estimate	Accuracy incentive	Advice imposed	# of advisors	Advice-taking task	*p*
Bailey et al. (2021a)a	57	19.5	students	54.4	Australia	90	credit	N	N	Y	1	rental prices	0.43
Bailey et al. (2021a)b	56	71.7	older adults	51.8	Australia	90	cash	N	N	Y	1	rental prices	0.58
Carbonell et al. (2019)	130	25.72	ns	70	Germany	67	mixed	Y	N	Y	1–20 or 250–350	physician ratings	0.54
De Hooge et al. (2014) Study 3	120	21.48	students	40	Netherlands	80	credit	Y	N	Y	1	coffee sales	0.51
De Hooge et al. (2014) Study 4	113	21.04	students	55.75	Netherlands	80	credit	Y	N	Y	1	coffee sales	0.68
De Hooge et al. (2014) Study 5	50	20.92	students	41.18	Netherlands	80	credit	Y	N	Y	1	coffee sales	0.61
De Wit, et al. (2017) Study 2	199	42	professionals	36.87	Chile & Netherlands	Mixed	cash	N	N	Y	1	vacation and furniture costs	0.40
De Wit et al. (2017) Study 3	72	21	ns	81.33	Western Europe	Mixed	mixed	N	N	Y	1	vacation and furniture costs	0.66
Fiedler et al., 2019, Study 1	48	ns	students	ns	Germany	67	mixed	Y	N	Y	1	health-related judgements	0.46
Gino and Moore (2007) Study 1	25	24	Students (86%)	42	US	91	cash	N	Y	Y	1	weight	0.59
Gino and Moore (2007) Study 2	40	25	students (90%)	38	US	91	cash	N	Y	N	1	weight	0.52
Gino (2008) Study 1	73	19–26	students	52	US	91	cash	N	Y	N	1	historical dates	0.50
Gino (2008) Study 2	88	26	students (93%)	51	US	91	cash	N	Y	Y	1	historical dates	0.47
Gino (2008) Study 3	168	23	students (88%)	42	US	91	cash	N	Y	N	1	historical dates	0.36
Gino and Schweitzer (2008) Study 1	109	20.28	students	50.46	US	91	cash	N	N	Y	1	weight	0.49
Gino and Schweitzer (2008) Study 2	107	21	students (95%)	49.53	US	91	cash	N	N	Y	1	weight	0.38
Gino et al. (2009) Study 1	135	52	community	54	US	91	cash	Y	N	Y	1	behaviour judgments	0.40
Gino et al. (2009) Study 2	106	24.00	students (73%)	51.00	US	91	cash	Y	N	Y	1	behaviour judgments	0.42
Gino et al. (2009) Study 3	123	26.00	students (54%)	57.00	US	91	cash	Y	N	Y	1	behaviour judgments	0.47
Gino et al. (2012) Study 1	83	21.00	students	54.90	US	91	cash	N	Y	N	1	weight	0.47
Gino et al. (2012) Study 2	127	21.10	students	58.27	US	91	cash	N	Y	Y	1	weight	0.33
Gino et al. (2012) Study 4	122	32.00	mixed	52.46	US	91	cash	N	N	Y	1	coins	0.32
Gino et al. (2012) Study 5B	189	21.00	students	62.43	US	91	cash	N	Y	Y	1	coins	0.38
Gino andet al. (2012) Study 5C	118	20.89	students	59.32	US	91	cash	N	Y	Y	1	coins	0.57
Gino et al. (2012) Study 6	139	20.00	students	41.73	US	91	cash	N	Y	Y	1	coins	0.47
Haran and Shalvi (2020) Study 2	221	37.42	MTurk	51.58	US, Canada, UK	Mixed	cash	N	Y	Y	1	number problem	0.50
Haran and Shalvi (2020) Study 3	350	35.15	MTurk	46.94	ns	ns	cash	N	Y	Y	1	coins	0.51
Haran and Shalvi (2020) Study 4	200	36.60	MTurk	53.50	US, Canada, UK, Australia	Mixed	cash	N	Y	Y	1	number problem	0.43
Haran and Shalvi (2020) Study 5	205	36.33	MTurk	50.71	US	91	cash	N	Y	Y	1	number problem	0.41
Häusser et al. (2016)	96	25.60	students	64.58	Germany	67	cash	N	N	Y	1	distances	0.39
Hofheinz et al. (2017)a	28	38.98	community	77.14	Germany	67	cash	N	N	Y	1	age and distance	0.24
Hofheinz et al. (2017)b	28	38.98	depressed patients	77.14	Germany	67	none	N	N	Y	1	age and distance	0.34
Hütter and Ache (2016) Study 1	35	23.00	students	71.43	Germany	67	mixed	N	Y	Y	1	calories	0.11
Hütter and Ache (2016) Study 2	44	26.48	students	68.18	Germany	67	mixed	N	Y	Y	1 to 20	calories	0.27
Hütter and Ache (2016) Study 3	58	21.10	students	77.59	Germany	67	mixed	N	Y	Y	1 to 20	calories	0.19
Hütter and Fiedler (2019) Study 1	52	24.12	students	68.85	Germany	67	credit	N	N	Y	1	historical dates	0.57
Hütter and Fiedler (2019) Study 2	45	21.86	students	58.89	Germany	67	mixed	N	N	Y	1	historical dates	0.43
Hütter and Fiedler (2019) Study 3	60	23.51	students	77.78	Germany	67	mixed	N	N	Y	1	historical dates	0.40
Hütter and Fiedler (2019) Study 4	30	22.19	students	72.22	Germany	67	mixed	N	N	Y	1	historical dates	0.37
Kadous et al. (2013) Study 1	88	ns	professionals	ns	US	91	none	Y	N	Y	1	discount rate	0.35
Kaliuzhna et al. (2012)a	30	37.30	patients with schizophrenia	26.67	France	71	none	N	N	Y	1	historical dates	0.54
Kaliuzhna et al. (2012)b	30	39.60	community	43.33	France	71	none	N	N	Y	1	historical dates	0.39
Kausel et al. (2015) Study 1	278	19.01	students	55.04	US	91	credit	N	N	Y	1	general knowledge	0.54
Kausel et al. (2015) Study 2	271	38.00	MTurk	58.00	US	91	cash	N	N	Y	1	general knowledge	0.38
Kausel et al. (2015) Study 3	150	37.00	MTurk	61.00	US	91	cash	N	N	Y	1	general knowledge	0.42
Kausel et al. (2015) Study 4	124	ns	students	55.65	US	91	credit	N	N	Y	1	weight	0.54
Kim et al. (2020) Study 1	60	21.95	students	21.25	South Korea	18	cash	Y	Y	Y	1	forecasting	0.45
Kim et al. (2020) Study 2	26	21.00	students	37.08	South Korea	18	cash	Y	Y	Y	1	forecasting	0.59
Kim et al. (2020) Study 3	15	21.95	students	21.67	South Korea	18	cash	Y	Y	Y	1	forecasting	0.25
Larson et al. (2020)	48	ns	students	ns	US	91	credit	N	Y	Y	1 or 2	historical dates	0.25
Logg et al. (2019) Study 1A	202	28*	online platform	44.55	US	91	ns	N	Y	Y	ns	weight	0.38
Logg et al. (2019) Study 1B	215	31*	online platform	53.00	US	91	ns	Y	Y	Y	275	song rankings	0.29
Logg et al. (2019) Study 1C	286	37*	online platform	55.00	US	91	ns	Y	N	Y	48	romantic attraction	0.32
Logg et al. (2019) Study 2	154	21*	mixed	67.53	US	91	mixed	N	N	Y	1	weight	0.43
Logg et al. (2019) Study 4a	301	39.00	MTurk	51.16	US	91	cash	mixed	Y	Y	mixed	weight and forecasts	0.33
Logg et al. (2019) Study 4b	70	46.00	professionals	0.04	US	91	ipad	mixed	Y	Y	mixed	weight and forecasts	0.24
Meshi et al. (2012)	29	23.30	ns	58.62	Germany	67	cash	N	Y	Y	1	rental prices	0.56
Minson and Mueller (2012)	92	ns	ns	ns	US	91	cash	N	Y	Y	1 or 2	US geography, demography and commerce	0.32
Molleman et al. (2020)	95	35.8	MTurk	43.00	US	91	cash	N	Y	Y	3	number of animals	0.34
Molleman et al. (2021)a	16	10.00	children	37.50	Germany	67	cash	N	Y	Y	1	number of animals	0.47
Molleman et al. (2021)b	21	11.00	children	66.70	Germany	67	cash	N	Y	Y	1	number of animals	0.42
Molleman et al. (2021)c	18	12.00	children	44.40	Germany	67	cash	N	Y	Y	1	number of animals	0.43
Molleman et al. (2021)d	9	13.00	children	55.60	Germany	67	cash	N	Y	Y	1	number of animals	0.36
Molleman et al. (2021)e	13	14.00	children	23.10	Germany	67	cash	N	Y	Y	1	number of animals	0.35
Molleman et al. (2021)f	13	15.00	children	30.80	Germany	67	cash	N	Y	Y	1	number of animals	0.35
Molleman et al. (2021)g	13	16.00	children	46.20	Germany	67	cash	N	Y	Y	1	number of animals	0.23
Molleman et al. (2021)h	12	17.00	children	41.70	Germany	67	cash	N	Y	Y	1	number of animals	0.27
Molleman et al. (2021)i	9	18.00	mixed	44.40	Germany	67	cash	N	Y	Y	1	number of animals	0.38
Molleman et al. (2021)j	12	19.00	mixed	41.70	Germany	67	cash	N	Y	Y	1	number of animals	0.29
Molleman et al. (2021)k	10	20.00	mixed	50.00	Germany	67	cash	N	Y	Y	1	number of animals	0.21
Önkal et al. (2009) Study 1	76	19.00–22.00	students	45.00	Turkey	37	credit	Y	N	Y	1	stock forecasting	0.34
Önkal et al. (2017) Study 1	72	22.00	students	45.00–52.00%	Turkey	37	credit	Y	N	Y	1	stock forecasting	0.42
Önkal et al. (2017) Study 2	93	22.00	students	45.00–52.00%	Turkey	37	credit	Y	N	Y	1	stock forecasting	0.40
Önkal et al. (2017) Study 3	65	22.00	students	45.00–52.00%	Turkey	37	credit	Y	N	Y	1	stock forecasting	0.27
Önkal et al. (2017)Study 4	82	33.35	professionals	ns	Turkey	37	ns	Y	N	Y	1	stock forecasting	0.35
Prahl and Van Swol (2017)	157	18.00–23.00	students	75.00	US	91	credit	Y	Y	Y	1	forecasting tasks	0.63
Rakoczy et al. (2015)Study 1	39	4.92	children	46.15	US	91	no	Y	N	Y	1	how much to feed the animals	0.69
Rakoczy et al. (2015)Study 2	93	4.72	children	41.94	Germany	67	no	Y	N	Y	1	how much to feed the animals	0.75
Rees et al. (2013)Study 3B	658	31.34	ns	50.50	US	91	ns	N	N	N	up to 5	university tuition	0.34
Reyt et al. (2016)Study 2	158	37.14	online platform	48.00	US	91	cash	Y	Y	Y	1	management consulting	0.41
Ribeiro et al. (2020)a	347	ns	MTurk	62.00	US	91	ns	Y	N	Y	1	product launch recommendation	0.36
Ribeiro et al. (2020)b	137	ns	professionals	28.57	Brazil	38	ns	Y	N	Y	1	product launch recommendation	0.23
Sah et al. (2013)Study 1	184	18.00–35.00	students (> 90%)	51.00	US	91	cash	N	Y	Y	1	weight	0.69
Sah et al. (2013)Study 2	377	18.00–35.00	students (> 90%)	58.00	US	91	cash	N	Y	Y	1	weight	0.66
Scheunemann et al. (2020)a	79	30.10	MTurk/high psychotic-like	32.90	US	91	cash	mixed	N	Y	up to 4	age/number of friends/income	0.31
Scheunemann et al. (2020)b	1110	37.70	MTurk/low psychotic-like	58.40	US	91	cash	mixed	N	Y	up to 4	age/number of friends/income	0.28
Scheunemann et al. (2021)a	80	33.69	MTurk/high psychotic-like	36.25	US	91	cash	N	N	Y	up to 5	age	0.45
Scheunemann et al. (2021)b	1106	39.41	MTurk/low psychotic-like	58.86	US	91	cash	N	N	Y	up to 5	age	0.36
Schilbach et al. (2013)	27	28.26	ns	59.26	Germany	67	ns	N	N	Y	1	distances	0.33
Schul and Peri (2015) Study 2	60	ns	students	ns	Israel	54	mixed	N	Y	Y	1	coins	0.39
Schultze et al. (2015) Study 1	27	21.19	students	70.00	Germany	67	mixed	N	Y	Y	1	distances	0.24
Schultze et al. (2015) Study 2	31	24.35	students	58.00	Germany	67	mixed	N	Y	Y	1	calories	0.32
Schultze et al. (2015) Study 3	37	22.00	students	54.00	Germany	67	mixed	N	Y	Y	1	distances	0.35
Schultze et al. (2015) Study 4	97	21.32	students	65.00	Germany	67	mixed	N	Y	Y	1	distances	0.22
Schultze et al. (2015) Study 5	45	ns	students	ns	Germany	67	cash	N	Y	Y	1	historical dates	0.25
Schultze et al. (2015) Study 6	98	ns	students	ns	Germany	67	cash	N	Y	Y	1	calories	0.32
Schultze et al. (2017) Study 1	28	21.93	students	50.00	Germany	67	cash	N	Y	Y	1	distances	0.23
Schultze et al. (2017) Study 2	30	23.72	students	48.00	Germany	67	cash	N	Y	Y	1	distances	0.22
Schultze et al. (2017) Study 3	99	24.06	students	76.00	Germany	67	cash	N	Y	Y	1	distances	0.21
Schultze et al. (2017) Study 4	92	23.80	students	54.00	Germany	67	cash	N	Y	Y	1	distances	0.24
Schultze et al. (2018) Study 1	191	23.06	students	66.00	Germany	67	cash	N	Y	Y	1	distances	0.25
Schultze et al. (2018) Study 2	251	21.27	students	59.00	Germany	67	cash	N	Y	Y	1	distances	0.29
Schultze et al. (2018) Study 3	351	22.79	students	64.00	Germany	67	cash	N	Y	Y	1	distances	0.16
Schultze et al. (2019) Study 1	100	24.58	students	60.00	Germany	67	cash	N	Y	Y	up to 2	quantity	0.18
Schultze et al. (2019) Study 2	50	24.19	students	57.00	Germany	67	cash	N	Y	Y	1	quantity	0.38
Schultze et al. (2019) Study 3	50	23.63	students	59.00	Germany	67	cash	N	Y	Y	1	quantity	0.31
Schultze and Loschelder (2021)	195	23.03	students	63.00	Germany	67	ns	N	Y	Y	1	length of rivers	0.30
See et al. (2011) Study 2	63	20.03	students	36.00	US	91	cash	N	N	Y	1	university tuition	0.45
See et al. (2011)Study 3	254	26.50	community	61.00	US	91	ns	N	N	Y	1	coins	0.24
Tinghu et al. (2018)Study 1	102	23.27	students	50.00	China	20	cash	Y	N	Y	1	vocational decision task	0.43
Tinghu et al. (2018)Study 2	128	24.73	students	51.56	China	20	cash	Y	N	Y	1	vocational decision task	0.65
Tost et al. (2012)Study 1	107	21.00	students	49.53	US	91	cash	N	Y	Y	1	weight	0.33
Tost et al. (2012)Study 2	132	21.00	students	46.97	US	91	cash	N	Y	Y	1	coins	0.38
Tost et al. (2012)Study 3	199	23.00	students	66.33	US	91	cash	N	Y	Y	1	weight	0.40
Tost et al. (2012)Study 4	202	33.62	ns	56.00	US	91	cash	N	Y	Y	1	weight	0.41
Trouche et al. (2018)Study 1	76	33.70	MTurk	39.39	US	91	cash	N	N	Y	1	life expectancy by country	0.23
Trouche et al. (2018)Study 2	84	36.40	MTurk	0.44	US	91	cash	N	N	Y	1	life expectancy by country	0.20
Trouche et al. (2018)Study 3	34	32.60	MTurk	42.00	US	91	cash	N	N	Y	1	life expectancy by country	0.17
Trouche et al. (2018)Study 4	37	35.00	MTurk	26.00	US	91	cash	N	N	Y	1	life expectancy by country	0.16
Tzini and Jain (2018)Study 1B	101	29.91	MTurk	38.61	India	48	cash	N	Y	Y	1	weight	0.32
Wang and Du (2018) Study 1	50	21.48	students	64.00	China	20	notebook	N	Y	Y	1	coins	0.24
Wang and Du (2018) Study 2	94	23.02	students	71.28	China	20	notebook	N	Y	Y	1	coins	0.41
Wang and Du (2018) Study 3	104	21.01	students	59.62	China	20	notebook	N	Y	Y	1	coins	0.36
Wanzel et al. (2017)	79	24.89	students	66.00	ns	ns	ns	N	Y	Y	3	calories	0.39
Yaniv and Kleinberger (2000) Study 1	25	ns	students	ns	Israel	54	mixed	N	Y	Y	1	historical dates	0.29
Yaniv and Milyavsky (2007) Study 2	75	ns	ns	ns	Israel	54	ns	N	Y	Y	2	historical dates	0.35
Yoon et al. (2021) Study 2A	896	37.89	MTurk	39.50	ns	ns	cash	N	N	Y	1	object weight	0.33
Yoon et al. (2021) Study 2B	494	37.77	MTurk	44.33	ns	ns	cash	N	N	Y	1	object weight	0.36
Zhang and North (2020) Study 4	429	36.99	MTurk	58.00	ns	ns	cash	Y	N	Y	1	car purchase	0.71

* refers to median, N = No, Y = Yes, ns = not specified. Students = university students. We referred to https://www.hofstede-insights.com/product/compare-countries/ to determine Individualism ratings. Number of advisors also refers to pieces of advice and represents the number in a single trial. The pooled weight of advice (*p*) refers to the raw proportion of advice-taking and was calculated as described in the meta-analytic approach section. Hofheinz et al. (2017) provided mean age and percent female averaged across two samples that did not differ significantly in age or percent of females.

**Table 2:** Summary of Predictor Variables

Predictor name	Definition	Operationalisation
Perceived advice quality	Information about the advisor(s) that suggests the advice is higher or lower in quality than the judge’s estimate	High, Neutral, Low
Uncertainty of estimate	The estimate is subjective and uncertain, or there is one objectively correct and certain estimate	Yes (uncertain), No (objective)
Actual advice accuracy	The advice closely estimates an objectively correct answer	Yes, No, NA if the estimate is uncertain
Accuracy incentive	The judge is advised that they will be rewarded if their estimates are accurate	Yes, No
Participation payment	Any reimbursement for participant time that is not related to performance on the judge-advisor task or any other task	Cash, Course credit, Mixed, Other (e.g., ipad)
Advice imposed	On each decision trial the judge is advised of at least one estimate without needing to solicit that advice	Yes, No
Number of advisors	A single piece of advice is the average estimate of more than one advisor	Yes (more than 1 advisor), No (1 advisor)
Age	Mean age of the sample of judges	Mean
Percent female	Proportion of female judges in the sample	% female
Culture	Degree of individualism in the country where the judges reside	% individualism
Student status	The sample of judges are either university students or non-students	Student, Non-student
Confidence	The judge’s average pre-advice confidence rating converted to a standardised scale	0 to 1; larger scores indicate greater confidence

Refer to Figs. 4, S1 and S2 for a list of studies that included high, neutral, and low perceived advice quality, respectively. Refer to Table 1 for representative sources for each predictor

## Data Availability

Data and code are accessible at the Open Science Framework (https://osf.io/atz6y/?view_only=a5e435f0b5de42a286736725a11bb58d).
