# Supplementary material for: A meta-analysis of the weight of advice in decision-making
Source: Curr Psychol. Author manuscript; Available in PMC 2024 Dec 20. (PMC11661855; doi:10.1007/s12144-022-03573-2)
Supplement: Supplementary Information [file NIHMS1996665-supplement-Supplementary_Information.docx]

**Supplementary Information**

**Coding Perceived Advice Quality**

Advisors were coded as being perceived to provide high quality advice if described as having more knowledge about the problem than the participant (i.e., Sah et al., 2013), or if the advice was framed as coming directly from the experimenter (Tinghu et al., 2018), noting that there was no sham advice provided. When participants believed advice was the average estimate of more than one previous participant (i.e., Carbonell et al., 2019; Larson et al., 2020; Logg et al., 2019), we coded it as high quality based on the statistical principle that the aggregation of imperfect estimates improves accuracy (Morin et al., 2021). Perceived quality was also coded as high if participants were told that the advisor based the advice on analytics (Ribeiro et al., 2020), or spent a lot of time analysing the task (Kadous et al., 2013). We coded perceived advisor expertise as high regardless of whether an advisor was simultaneously described as low in confidence (Sah et al., 2013). This was because it is possible to be accurate and an expert while also lacking confidence. Indeed, the correlation between an advisor’s accuracy and confidence can be low (Bonaccio & Dalal, 2006).

We rated Zhang and North’s (2020) younger, older, and same-age advisors all as neutral in perceived quality of advice because we did not have evidence to suggest that the participants would perceive these advisors differently. When no information was provided to participants about the source of advice it was coded as neutral for perceived advice quality (e.g., Yaniv & Milyavsky, 2007). However, when a study did not explicitly state that participants were provided with no information about the advisor, the study was excluded (e.g., Wang & Du, 2018, Study 2; Yaniv & Kleinberger, 2000, Study 1). When participants were told that advice was from another participant, or a previous participant dyad (Minson & Mueller, 2012), the perceived advice quality was coded as neutral. When participants were also told that the previous participant performed in the 50th percentile for accuracy (i.e., an average performer), their perceived advice quality was coded as neutral (Tost et al., 2012, Study 3, Study 4). An advisor was coded as providing low quality advice if their estimate was based on intuition (Ribeiro et al., 2020), or if they were described as having not spent a lot of time analysing the task (Kadous et al., 2013). According to Ribeiro et al. (2020), “In the collective imaginary, intuition still has, to some extent, mystical, magical and spiritual connotations, weakening its power as a valid source of judgment.” (p. 17). Advisors described as accurate in 3 out of 6 previous estimates (or 50% of the time) were coded as providing low quality advice, while those described as accurate in 4 out of 6 estimates were coded as neutral, and 5 out of 6 was coded as high quality (Gino et al., 2012, Study 5C; Haran & Shalvi, 2020). Advisors described as having a conflict of interest whereby they have an incentive to provide bad advice were rated as low quality (Gino et al., 2012, Study 6).

Tost et al. (2012) manipulated feelings of power in the participant and differentiated between competitive versus cooperative mindset. We coded perceived advice quality based on the description of the advisor rather than the manipulation of the participant feelings of power or mindset. Similarly, Kadous et al. (2013) manipulated feelings of social connection with the advisor, and we coded perceptions of the advisor rather than the manipulation of participants’ feelings.

We excluded 18 effect sizes (out of 346; 5.2%) where feedback provided an opportunity to learn about advice quality across the course of the task (i.e., Prahl & Van Swol, 2017, Yoon et al. 2021). Five effect sizes (out of 346; 1.4%) were excluded because perceived advice quality was confounded with advisor motives. That is, the participant expected mostly accurate advice from an advisor who they knew would occasionally intentionally mislead them (see Haran & Shalvi, 2020, Study 2, Study 3, Study 4). Eight effects (2.3%) were excluded because information about the supposed advisor expertise was accompanied by additional instructions to evaluate initial estimate as too high or too low (Schultze et al., 2017, Study 4). Four effects (1.2%) were excluded because conflicting information was provided about advisor credibility/expertise (Önkal et al., 2017, Study 3). Two effects (0.6%) were excluded because the advisor was described as providing an estimate that was close to, or far from, the participant’s initial estimate (Wanzel et al., 2017). Thus, perceptions of advice quality depended on how accurate participants believed their own initial estimate to be, and there were no data to determine this. Based on conflicting evidence for both algorithm appreciation (Logg et al., 2019) and algorithm aversion (Prahl & Van Swol, 2017), six effects (1.7%) were excluded from perceived advisor quality analysis because participants were told that the advice was generated by an algorithm (Logg et al., 2019) or a statistical model (Önkal et al., 2009).

**Coding Uncertainty of the Estimate**

Estimations were coded as being of objectively correct versus subjective or uncertain values. For objective values there is one correct answer. For example, number of coins in a jar (e.g., Gino et al., 2012; Schul & Peri, 2015), or historical dates (e.g., Gino, 2008; Yaniv & Kleinberger, 2000). Whereas for subjective or uncertain values there is not one correct answer. Rather, the answer is determined by consensus. For example, stock forecasting (e.g., Önkal et al., 2009), online ratings recommending physicians (e.g., Carbonell et al., 2019), attractiveness ratings (e.g., Logg et al., 2019), sales forecasts (e.g., De Hooge et al., 2014), or behaviour judgments (e.g., Gino et al., 2009).

**Coding Actual Advice Accuracy**

Actual advice accuracy was determined with respect to deviations from the correct answer. Under conditions of subjectivity or uncertainty, advice could still be described as more or less accurate/reasonable (e.g., Prahl & Van Swol, 2017). However, we only analysed actual accuracy for studies that involved objectively correct answers. This was because, although a subjective estimate may be more or less reasonable than another, there is not one objectively accurate estimate. We did not code studies for accuracy if the advice accuracy was random (e.g., Gino & Moore, 2007, Study 1; Haran & Shalvi, 2020, Study 2 (6 effects; 1.7%), Study 3 (4 effects; 1.2%)), or if it was based on the participant’s initial estimate (Molleman et al., 2020; 4 effects; 1.2%). If advice was variable but only deviated a small amount from the accurate estimate, we coded it as accurate (e.g., Gino et al., 2012, Study 1, Study 2; Wang & Du, 2018, Study 1). Häusser et al. (2016) described the actual accuracy of advice as high (deviated from true value by 16%) and medium (deviated from true value by 55%). Following this approach, we coded this advice as accurate and inaccurate, respectively. If the actual advice was the mean estimate of a number of previous participants, it was coded as accurate (Gino et al., 2012, Study 5B, Study 6; Logg et al., 2019; Schultze & Loschelder, 2021; Wang & Du, 2018, Study 3; Yoon et al., 2021, Study 2A). Among the objective estimates, 136 effects were excluded because advice accuracy was not specified.

**Coding Other JAS-Level and Environment-Level Factors**

Three studies that paid participants via a cash lottery were combined with the studies paying cash for participation (i.e., De Wit et al., 2017, Study 2; Sah et al., 2013, Study 1, Study 2). If participants in a study were offered either cash or course credit, the payment was coded as ‘mixed’. A study that referred to participation in partial fulfilment of course requirement was coded as ‘credit’ (i.e., Larson et al., 2020). Studies were excluded if they did not refer to payments to participate, or lack thereof (e.g., Logg et al., 2019), with the exception of studies conducted on MTurk that we assumed offered cash payment for participation (e.g., Molleman et al., 2020; Scheunemann et al., 2021). Studies that offered rewards for participation such as iPads or notepads, and studies that were coded as ‘mixed’, were excluded from this analysis. Studies were coded as not offering performance-based accuracy incentives if there were none described (e.g., Kadous et al., 2013; Logg et al., 2019; Rees et al., 2013, Study 3B; Yoon et al., 2021, Study 2A, Study 2B). Studies were coded as imposing advice even if participants had the option of asking for more than one piece of advice (i.e., Scheunemann et al., 2020, 2021). Fourteen effects (4%) were excluded for the multiple advisor analysis because advice came from between one or more advisors (Carbonell et al., 2019; Hütter & Ache, 2016; Rees et al., 2013; Scheunemann et al., 2020, 2021).

**Coding Individual-Level (Judge) Characteristics**

If a sample included decision-makers from both individualist and collectivist cultures it was coded as ‘mixed’ (e.g., De Wit et al., 2017, Study 2). One sample was described as Western European and therefore could not be coded with a specific percentage for individualism (i.e., De Wit et al., 2017, Study 3). Five studies were coded as having ‘student’ samples when they noted that >85% of the sample were students (i.e., Gino, 2008, Study 2, Study 3; Gino & Moore, 2007, Study 1, Study 2; Gino & Schweitzer, 2008, Study 2). One study did not specify the type of participant (i.e., Meshi et al., 2012). We used median age (i.e., Logg et al., 2019, Study 1A, 1B, 1C, 2), or the mid-point of a small age range (i.e., Gino, 2008, Study 1, 19-26 years; Önkal et al., 2009, 19-22 years; Prahl & Van Swol, 2017, 18-23 years), when mean age was not available. For this analysis, we excluded samples when only a large age range was provided (i.e., 18-35 years; Sah et al., 2013, Study 1, Study 2). A number of studies did not describe percentage of females and thus were excluded for the respective analyses. We used the mid-point when a range of percent female was available (see Table 1).

**Supplementary Results**

**Overall Pooled Effect**

***Non-Predictors***

The judge’s pre-advice confidence ranged from 0.32 to 0.71 and did not predict weight of advice, *F*(1, 42) = 0.00, *p* = .986. Mean sample age ranged from 4.72 years to 71.7 years and did not predict the overall weight of advice, *F*(1, 293) = 0.89, *p* = .348. The type of sample (student (53.2% of effects) versus non-student (30.3% of effects)) did not predict weight of advice, *F*(1, 288) = 0.76, *p* = .384. The proportion of females in each study ranged from 0.04% to 81.33%, and did not predict weight of advice, *F*(1, 314) = 0.33, *p* = .567. Degree of individualism ranged from 18 to 91, and did not predict weight of advice, *F*(1, 301) = 0.93, *p* = .335. There was no influence of payment for participation (credit (*k* = 32) versus cash (*k* = 234)), *F*(1, 264) = 3.00, *p* = .085, whether advice was imposed (*k* = 333) versus optional (*k* = 13), *F*(1, 344) = 1.83, *p* = .177, or for multiple (*k* = 20) versus a single (*k* = 303) piece/s of advice, *F*(1, 321) = 0.98, *p* = .323.

***Predictors***

**Perceived Advice Quality.**  Perceived advice quality influenced weight of advice, *F*(2, 287) = 19.29, *p* < .001. The mean effect when perceived advice quality was neutral (*k* = 170) equaled 0.37, significantly deviating from zero, *t*(287) = 23.88, *p* < .001. The mean effect when perceived advice quality was high (*k* = 80) equaled 0.48, and this effect was significantly higher than the mean effect for neutral perceived advice quality, *t*(287) = 4.52, *p* < .001. The mean effect when perceived advice quality was low (*k* = 40) equaled 0.32, and this effect was not significantly different from the mean effect when perceived quality was neutral, *t*(287) = 1.63, *p* = .104.

**Estimate Uncertainty.** Objectivity influenced weight of advice, *F*(1, 336) = 11.61, *p* < .001. The mean effect for advice-taking where the estimate was subjective or uncertain (*k* = 94) was 0.47, and this deviated significantly from zero, *t*(336) = 19.12, *p* < .001. The mean effect for advice-taking for an objectively correct or certain answer (*k* = 244) was 0.37, which differed from the effect when the estimate was subjective, *t*(336) = 3.41, *p* < .001.

**Actual Advice Accuracy.** There was an influence of actual advice accuracy, *F*(1, 118) = 6.26, *p* = .014. The mean effect of inaccurate advice (*k* = 20) was 0.29, and this deviated significantly from zero, *t*(118) = 6.28, *p* < .001. The mean effect of accurate advice (*k* = 100) was 0.40, which differed from inaccurate advice, *t*(118) = 2.50, *p* = .014.

**Accuracy Incentive.** There was an influence of accuracy incentive, *F*(1, 344) = 4.63, *p* = .032. The mean weight of advice when there was no accuracy incentive (*k* = 158) equaled 0.42, and this differed from zero, *t*(344) = 22.94, *p* < .001. The mean effect when there was an accuracy incentive (*k* = 188) equaled 0.37, which differed from when there was no accuracy incentive, *t*(344) = 2.15, *p* = .032.

**Advice Perceived as Neutral Quality**

***Non-Predictors***

There were no effects of the judge’s pre-advice confidence, *F*(1, 29) = 0.07, *p* = .800, age, *F*(1, 153) = 1.51, *p* = .221, percent of females, *F*(1, 154) = 0.17, *p* = .681, individualism, *F*(1, 161) = 1.25, *p* = .265, or student (*k* = 93) versus non-student (*k* = 38) sample, *F*(1, 129) = 0.33, *p* = .565. Weight of advice was not predicted by accurate (*k* = 45) versus inaccurate (*k* = 10) advice, *F*(1, 53) = 0.42, *p* = .518, advice choice (imposed (*k* = 157) vs optional (*k* = 13)), *F*(1, 168) = 3.17, *p* = .077, or single (*k* = 136) versus multiple (*k* = 14) advisors, *F*(1, 149) = 1.50, *p* = .223.

***Predictors***

**Estimate Certainty.** Weight of advice was influenced by estimating objectively correct (*k* = 131) versus subjective or uncertain (*k* = 33) values, *F*(1, 162) = 34.81, *p* < .001. The mean effect for advice-taking for a subjective or uncertain estimate was 0.55, and this significantly differed from zero, *t*(162) = 18.20, *p* < .001. The mean effect for advice-taking while estimating an objectively correct value was 0.35, which deviated significantly from estimates of subjective or uncertain values, *t*(162) = 5.90, *p* < .001.

**Accuracy Incentive.** Weight of advice was influenced by accuracy incentive (*k* = 98) versus no incentive (*k* = 72), *F*(1, 168) = 5.34, *p* = .022. The mean effect for advice-taking when there is no accuracy incentive is 0.42, and this significantly differs from zero, *t*(168) = 18.55, *p* < .001. The mean effect for advice-taking when there is an accuracy incentive is 0.35, which deviated significantly from when there was no accuracy incentive, *t*(168) = 2.31, *p* = .022.

**Participation Payment.** Weight of advice was influenced by participation payment (cash (*k* = 116) versus course credit (*k* = 17)), *F*(1, 131) = 7.35, *p* = .008. The mean effect for advice-taking when cash was paid was 0.38, and this significantly differed from zero, *t*(131) = 22.16, *p* < .001. The mean effect for advice-taking when course credit was provided was 0.51, which deviated significantly from when cash was paid, *t*(131) = 2.71, *p* = .008.

**Advice Perceived as High Quality**

The summary effect when perceived advice quality was high (*k* = 80) equaled 0.48, 95% CI [0.43, 0.53], *t*(79) = 19.25, *p* < .001 (see Figure S1). A boxplot identified no outlier effect sizes.

**Figure S1**

*Forest Plot of the Weight of Advice in Response to Advisors Perceived to Provide High Quality Advice*


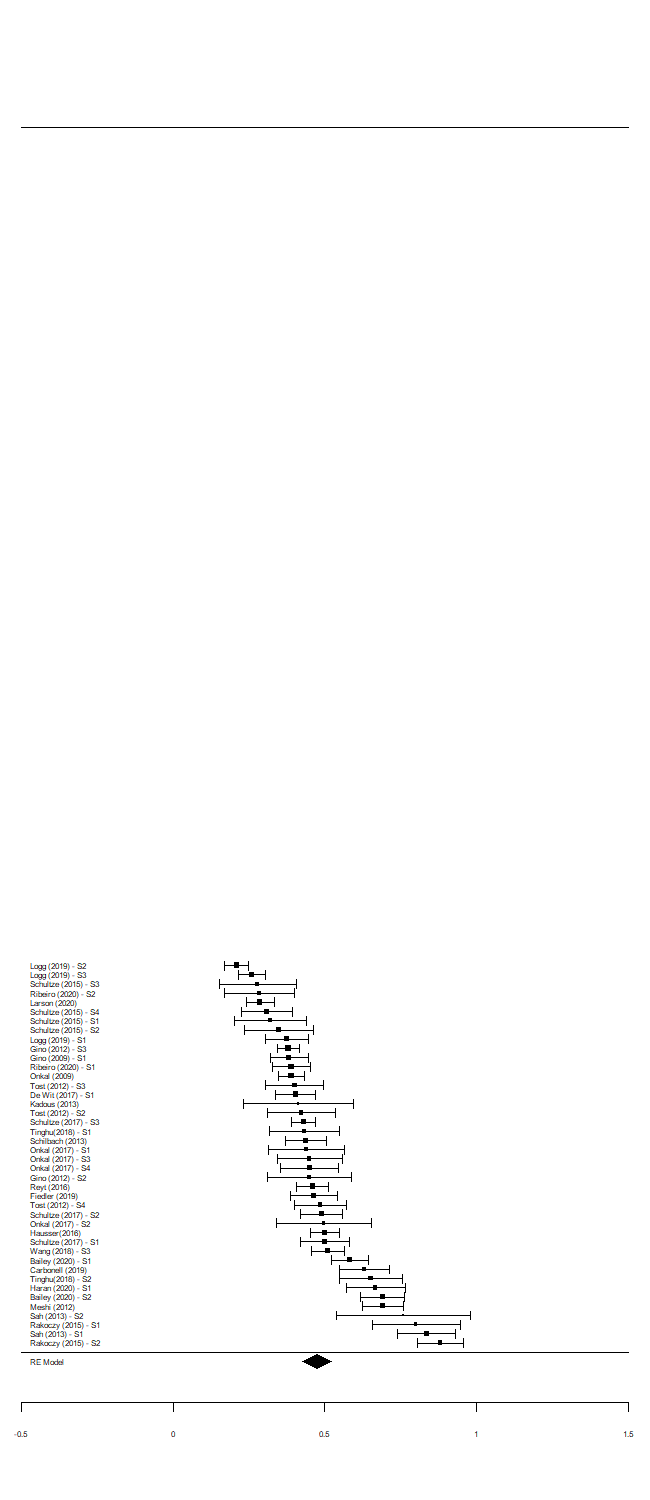


*Note*. The diamond represents the pooled weight of advice proportion. Each effect size and 95% confidence interval (error bar) represents an independent sample (*s* = 42). For articles with multiple independent samples, the effect size for each sample (S1, S2, etc.) is reported separately. Where a sample contributed more than one effect, the pooled effect, accounting for dependency between effects, is represented.

The original three-level model was a better fit than the two-level model in which level 2 (within-study variance) was not modelled (*p* < .0001), and the two-level model where level 3 (between-study variance) was fixed at zero (*p* = .0013). Consequently, there was significant variability between effect sizes within- and between-studies, and the estimated variance components were *τ*^2^_Level2_ *=* 0.013 and *τ*^2^_Level3_ *=* 0.019, respectively. Of the total variance, 3.49 percent was attributed to variance at level 1 (i.e., sampling variance); 58.11 percent was attributed to level 2 (i.e., within-study variance); and 38.40 percent was attributed to level 3 (i.e., between-study variance). We therefore extended our model to examine potential predictors.

***Non-Predictors***

Weight of advice was not predicted by mean age, *F*(1, 59) = 0.64, *p* = .429, percentage of females, *F*(1, 70) = 0.02, *p* = .900, individualism, *F*(1, 72) < 0.01, *p* = .967, or student (*k* = 47) versus non-student (*k* = 25) sample, *F*(1, 70) = 1.62, *p* = .207, objectively correct (*k* = 43) versus subjective/uncertain (*k* = 37) values, *F*(1, 78) = 0.32, *p* = .575, accuracy incentive (*k* = 37) versus no incentive (*k* = 43), *F*(1, 78) = 0.02, *p* = .882, participation payment (cash (*k* = 54) versus credit (*k* = 6)), *F*(1, 58) = 0.83, *p* = .366. All advice from advisors perceived to provide high quality advice was imposed, and only two effect sizes related to inaccurate advice, four to multiple advisors, and three related to pre-advice confidence ratings. These factors could therefore not be tested as potential predictors.

**Advice Perceived as Low Quality**

The summary effect when the advisor was perceived to provide low quality advice (*k* = 40) equaled 0.32, 95% CI [0.25, 0.38], *t*(39) = 9.57, *p* < .001 (see Figure S2). A boxplot identified no outlier effect sizes.

**Figure S2**

*Forest Plot of the Weight of Advice in Response to Advisors Perceived to Provide Low Quality Advice*


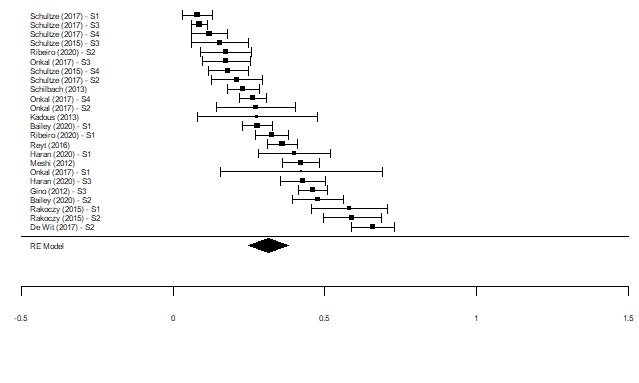


*Note*. The diamond represents the pooled weight of advice proportion. Each effect size and 95% confidence interval (error bar) represents an independent sample (*s* = 24). For articles with multiple independent samples, the effect size for each sample (S1, S2, etc.) is reported separately. Where a sample contributed more than one effect, the pooled effect, accounting for dependency between effects, is represented.

The original three-level model was a better fit than the two-level model in which level 2 (within-study variance) was not modelled (*p* < .0001), as well as the two-level model where level 3 (between-study variance) was fixed at zero (*p* = .0018). Consequently, there was significant variability between effect sizes within- and between-studies, and the estimated variance components were *τ*^2^_Level2_ *=* 0.018 and *τ*^2^_Level3_ *=* 0.010, respectively. Of the total variance, 2.86 percent variance was attributed to level 1 (i.e., sampling variance); 33.65 percent was attributed to level 2 (i.e., within-study variance); and 63.49 percent was attributed to level 3 (i.e., between-study variance). We therefore extended our model to examine potential predictors.

***Non-Predictors***

Weight of advice was not predicted by age, *F*(1, 30) < .01, *p* = .960, percentage of females, *F*(1, 33) = 0.07, *p* = .799, or individualism, *F*(1, 30) = 2.29, *p* = .141, or whether the sample included a student (*k* = 16) versus non-student (*k* = 16) sample, *F*(1, 30) = 3.60, *p* = .067. There was no effect of objectively correct (*k* = 25) versus subjective or uncertain (*k* = 15) estimates, *F*(1, 38) = 0.11, *p* = .745, or accuracy incentive (*k* = 20) versus no accuracy incentive (*k* = 20), *F*(1, 38) = 1.72, *p* = .197. Only four effect sizes related to payment with course credit, and only three included a judge’s pre-advice confidence rating. Similarly, there were no studies where the advisor was perceived to provide low quality advice that offered inaccurate advice, or advice from more than one advisor, or optional advice.
